# The Vindication of Voluntaryism

**Published:** 1924-11

**Authors:** 


THE
### HOSPITAL and HEALTH REVIEW

### THE VINDICATION OF VOLUNTARYISM.

No more remarkable indication of the vitality of the voluntary hospital system could be imagined than that which is to be found in the fifth annual report of the Joint Council of the Order of St. John and the British Red Cross Society on "The Voluntary Hospitals in Great Britain," which has been issued during the past month. Unhappily Sir Napier Burnett, the originator of this exceedingly valuable series, is no longer with us, and the report has been prepared by his successor, Dr. Kay Menzies. In his introductory remarks Sir Arthur Stanley pays a high tribute, which will be echoed by every lover of hospitals, to the labour and self-sacrifice which Sir Napier Burnett devoted to work that is now beginning to bear such splendid fruit. The report naturally takes no account of the London hospitals, but it deals with 93 per cent. of those in the provinces and with a rather larger proportion of those in Scotland, and Sir Arthur Stanley is, if anything, unduly modest when he says that its figures "prove that the voluntary system is holding its own." It is, indeed, doing much more than that. Not only did the combined income of these 705 hospitals reach the splendid total of £7,689,000, but it showed a surplus of £213,000 of ordinary income over ordinary expenditure. The improvement, as compared with the immediate past, is thrown into high relief by the figures of the two previous years. In 1921 the ordinary expenditure of the hospitals exceeded their ordinary income by £419,000, and in 1922 the balance was still £75,000 on the wrong side. The past year was, therefore, nearly £300,000 better than its predecessor, and it is important to observe that only a little more than 8 per cent. of the year's income was derived from the Public Services or the Voluntary Hospitals Commission. That is to say, well over seven millions were raised by voluntary offerings in one form or another. The greater aggregate of patients is to a very large extent explained by the larger number of hospitals taken into account, and we may reasonably expect that the small percentage of institutions that have failed to respond to the request for figures and other data will speedily remove from themselves what is really a reproach.

The labour of preparing a report of this comprehensive and analytical character must, in any case, be great, but Dr. Kay Menzies points out that it is far heavier than it need be if hospitals generally would unify their methods of account-keeping. Hospitals are now rapidly emerging from the individualistic stage and entering upon one in which system and co-ordination are essential. A vast amount of cost and effort could be saved if it were easier for one hospital to benefit by the experience of another in somewhat comparable circumstances, and in a paper appended to the report, Mr. J. E. Stone, the accountant of St. Thomas's Hospital, insists, as he has already insisted in our own columns, upon the paramount importance of scientific account-keeping. The voluntary system must show that it is as efficient on the business as on the medical, surgical, and preventive sides. Nor is what may be called the efficiency of the moment enough to satisfy those who are careful for the future. We have to look ahead and try to form some notion of what the needs of the hospitals and their potential patients may be a decade or two hence. Something definite is already being done in this direction. At the request of the Minister of Health, the Voluntary Hospitals Commission is engaged upon an inquiry into "the extent of the additional Voluntary Hospital accommodation required in Great Britain, and the best means of providing and maintaining it." So far so good, but Sir Arthur Stanley suggests, and he has the support of the British Hospitals Association and the Joint Council, that this is not enough. He asks that the scope of the enquiry should be extended, and that investigation should be made into the general need of hospital accommodation, the directions in which that need is felt, and the possibility of supplying that accommodation in part, at all events, by better co-ordination and co-operation. It is not enough to know how many beds are required per thousand of the population. It is essential that we should also know for what they are required and how far, if at all, they fall short in any particular category. It would then be possible to map out a comprehensive and elastic plan which would enable means to be adjusted to ends over a considerable series of years. At present we are living from hand to mouth, and so closely absorbed in providing for immediate individual needs that we have hardly begun to consider the imperative necessity of atmosphere and vision. For to-day the hospitals appear to be safe, but it is essential that we should legislate for to-morrow, and be prepared with plans for widening and broadening the basis upon which they stand, and placing them in a definite relation to what may be required of them in a not distant future.

In the meantime there are obvious opportunities lying ready to hand for that co-operation which is essential for making the best of the existing system. The responsibilities of the Public Health Authorities are certain steadily to increase, and much could be done to prepare for that extension by co-operation between them and the voluntary hospitals. There will be general agreement with Dr. Kay Menzies when he says that hospital boards should acquaint themselves with the general needs of their area by conferring with the Public Health Authorities and the general practitioners who, between them, know much that can be only partially apprehended by the hospitals. Such conferences would show whether and in what directions they can usefully co-operate in the provision of facilities for the prevention and treatment of disease. Already there is much valuable co-operation in the treatment of tuberculosis and other diseases, in the extension of maternity and child-welfare work, and in cognate directions. Much of this joint effort would necessarily be directed to that prevention of disease which will be the great scientific triumph of the future. It is only by becoming a healthier and more robust people that we can keep out of hospital and so circumscribe the demands upon the hospital system that there will be little room for the ill-informed demand for costly bureaucratic interference.

